# Do Human iPSC-Derived Cardiomyocytes Cultured on PLA Scaffolds Induce Expression of CD28/CTLA-4 by T Lymphocytes?

**DOI:** 10.3390/jfb13010006

**Published:** 2022-01-11

**Authors:** David Sergeevichev, Victor Balashov, Victoria Kozyreva, Sophia Pavlova, Maria Vasiliyeva, Alexander Romanov, Elena Chepeleva

**Affiliations:** 1 E. Meshalkin National Medical Research Center, The Ministry of Health of the Russian Federation, 15, Rechkunovskaya Str., 630055 Novosibirsk, Russia; vs_kozyreva@meshalkin.ru (V.K.); sonpavlova@gmail.com (S.P.); vasilievam@yandex.ru (M.V.); abromanov@mail.ru (A.R.); amareza@mail.ru (E.C.); 2 Institute of Theoretical and Experimental Biophysics, Russian Academy of Sciences, 3, Institutskaya Str., Puschino, 142290 Moscow, Russia; balachoff@yandex.ru; 3 Federal Research Center Institute of Cytology and Genetics, Siberian Branch of Russian Academy of Sciences, 10, Ac. Lavrentiev Ave., 630090 Novosibirsk, Russia; 4 Zelman Institute for the Medicine and Psychology, The Novosibirsk State University, 1, Pirogov Str., 630090 Novosibirsk, Russia; 5 Research Institute of Clinical and Experimental Lymphology, Branch of the Federal Research Center Institute of Cytology and Genetics, Siberian Branch of Russian Academy of Sciences, 2, Timakova Str., 630060 Novosibirsk, Russia

**Keywords:** graft rejection, iPSC, differentiation, cardiomyocytes, electrospinning, CD28, CTLA-4, immune response

## Abstract

Many research groups have developed various types of tissue-engineered cardiac constructs. However, the immunological properties of such artificial tissues are not yet fully understood. Previously, we developed microfiber scaffolds carrying human iPSC-derived cardiomyocytes (hiPSC-CM). In this work, we evaluated the ability of these tissue-engineered constructs to activate the expression of CD28 and CTLA-4 proteins on T lymphocytes, which are early markers of the immune response. For this purpose, electrospun PLA microfiber scaffolds were seeded with hiPSC-CM and cultured for 2 weeks. Allogeneic mononuclear cells were then co-cultured for 48 h with three groups of samples: bare scaffolds, pure cardiomyocyte culture and tissue-engineered constructs, followed by analysis of CD28/CTLA-4 expression on T lymphocytes using flow cytometry. PLA scaffolds and concanavalin A stimulation (positive control) statistically significantly increased CD28 expression on CD4^+^ T cells (up to 61.3% and 66.3%) CD8^+^ T cells (up to 17.8% and 21.7%). CD28/CTLA-4 expression was not increased when T lymphocytes were co-cultured with cardiac tissue-engineered constructs and iPSC-CM monolayers. Thus, iPSC-CM in monolayers and on PLA microfiber scaffolds did not induce T cell activation, which suggests that such cardiac constructs would not be a cause of rejection after implantation.

## 1. Introduction

Functional repair of damaged myocardium remains a challenging task for cardiac tissue engineering [1]. One promising approach to solve this problem is the use so-called cardiac tissue-engineered constructs, the purpose of which is to deliver new cells to a damaged area [2]. Such constructs can consist of cellular and biomaterial components loaded with bioactive molecules [3]. However, an important issue that still needs to be addressed is the host`s immune response and inflammation, both of which damage cells and scaffolds and can cause fibrosis.

For a long time, tissue from rats or other animals was used as a source of cardiomyocytes. However, these cells were only use in research and could not be applied for treatment because of insufficient quantity and strong immune reactions associated with transplantation. This problem has been solved with the emergence of so-called cardiomyocytes differentiated from induced pluripotent stem cells that have been actively developed in the last decade [4]. iPSCs are a unique source for obtaining a sufficient number of cardiomyocytes (CM) when creating cardiac tissue-engineered constructs. To date, there are a number of protocols for the directed differentiation of induced human and animal pluripotent stem cells into cardiomyocytes [5,6].

However, it is inefficient to use hiPSC-CM alone in the development of myocardial damage treatment infarction. It has been shown that direct intramyocardial transplantation of cardiac cells without a matrix leads to poor survival and an insufficient fixation at the injection site [7]. This is due to the fact that cardiomyocytes are anchorage-dependent cells and must be cultured on a substrate for long-term survival. To overcome the problems of poor survival and engraftment, it is useful to use two component tissue-engineered cardiac tissues consisting of a cellular component grown on a polymer matrix.

Typically, scaffolds for tissue engineering are made from natural and synthetic polymers that are further modified to obtain morphology and characteristics corresponding to cardiac tissue. These include polyglycolic acid (PGA), poly(L)-lactic acid (PLA), poly (DL)-glycolate (PLGA) and polyvinyl alcohol or their derivatives [8,9,10]. Among the methods of processing biomaterials, electrospinning occupies an important place since it allows one to obtain fibers with the size of natural extracellular matrix fibers. Other advantages are the ability to reproduce the complex microstructure of natural heart tissues, good reproducibility and the possibility of functionalization. [11]. However, the electrospun scaffold itself can activate the host immune response, which should be taken into account.

The use of polymer fiber matrices as cellular substrates can promote the proper development of cardiac tissue, provide its mechanical properties and provoke functional electrophysiological fusion of donor cardiomyocytes and recipient tissues [12]. Based on our earlier results, PLA scaffolds were selected for the creation of cardiac constructs and the study of their immunological properties [13].

The development of methods for transplantation of cells and cell-carrying tissue-engineered constructs (TEC) includes the study of donor cell survival and the associated activation of the T cell response. Prevention of immune-mediated inflammation and subsequent post-transplant degeneration of TEC remains relevant. The recipient’s immune system recognizes the donor’s MHC class I antigens located on almost all nucleated cells and activates the immune response. Two types of T cells, CD4 and CD8, are most involved in the rejection reaction. Activated CD4^+^ T cells proliferate, secreting various cytokines, growth factors and activation factors for CD8^+^ cytotoxic T cells, B cells and macrophages, which cause graft destruction [14]. Achievement of the required level of immune response occurs after transmission of an additional signal from coactivation molecules CD28 and CTLA-4. This results in further activation–differentiation or anergy-apoptosis of T and B lymphocytes. Control of these mechanisms and the creation of hypoimmune cell products can become a clinical approach in modulating allogeneic tolerance in cell transplantation [15,16]. Therefore, to assess the biocompatibility of TECs carrying hiPSC- CM, we performed experiments to investigate the ability of TECs to induce the expression of regulatory markers CD28 and CTLA-4 on T-lymphocytes in vitro.

## 2. Materials and Methods

### 2.1. Generation of Cardiomyocytes

A human iPSC (iMA-1L cell line, ICG SB RAS, Novosibirsk, Russia) was applied for directional differentiation into cardiomyocytes [17]. hiPSC was cultured on an LDEV-Free Matrigel™ hESC-qualified matrix (Corning Inc., Corning, NY, USA) in Essential-8 medium (ThermoFisher Scientific, Waltham, MA, USA). Differentiation was carried out in accordance with a previously published protocols [6,18] based on the activation of the WNT pathway using CHIR99021 (StemRD, Burlingame, CA, USA) for 48 h and subsequent inhibition with IWP2 (Merck KGaA, Darmstadt, Germany) in RPMI-1640 (Lonza, Köln, Germany) with B27-supplement (ThermoFisher Scientific, USA) without insulin. The appearance of spontaneously contracting areas was observed on days 8–10 of differentiation. On days 14–18 of differentiation, the cells were dissociated using TrypLE Express (ThermoFisher Scientific, USA) and transferred into 6-well plates coated with Matrigel™ (Corning, USA) in RPMI-1640 medium supplemented with 20% embryonic bovine serum (Autogene Bioclear, Calne, UK) and 10 µM Y-27632 supplement (StemRD, USA). Two days after the transfer and within one week, metabolic cell selection was carried out to purify the population of cardiomyocytes. Composition of the metabolic selection medium: RPMI-1640 without D-glucose (ThermoFisher Scientific, USA), 213 μg/mL L-ascorbic acid 2-phosphate (Sigma-Aldrich, Burlington, MA, USA), 500 µg/mL recombinant human albumin expressed in Oryza sativa (Sigma-Aldrich, USA) and 5 mM DL-sodium lactate L4263 (Sigma-Aldrich, USA). Polymeric microfiber matrices were colonized with 25-day-old hiPSC-CM. A part of enriched hiPSC-CM was cultured up to 40 days until the immunological studies started.

### 2.2. Production of Polymer Matrices and Seeding

Electrospinning was performed using a Nanon-01 device (MECC Corp., Fukuoka, Japan) according to Chepeleva E.V. et al. [13]. To obtain microfiber matrices, a 25 mg/mL solution of poly(L)-lactic acid (MW ~ 700 kDa) in hexafluoroisopropanol (all Sigma-Aldrich, USA) was used. The solution was fed using a 3 mL syringe with a 24 G needle at a rate of 0.5–2 mL/h. Fibers were sprayed onto polydimethylsiloxane rings (Dow Сhemical, Midland, MI, USA) laid on a flat electrospinning collector. The distance from the needle tip to the collector was 10 cm. The voltage between the needle and the collector was 5 to 7 kV. The electrospinning process was continued until a dense and stable layer of fibers was obtained. Prepared samples were removed from the rings and sterilized on all sides with UV irradiation in a laminar flow hood for one hour. Sterilized matrices were transferred in Petri dishes and stored under sterile conditions for no more than 3 days. Prior to hiPSC-CM colonization, polymer matrices were treated with Matrigel™ (Corning, USA) according to the manufacturer′s recommendations. Cells were seeded on the matrix surface with a density of 150,000/cm^2^. The formation of CM layer was completed on the second day. By the beginning of the immunological studies, the age of the CM on TECs reached 40 days.

### 2.3. Immunological Studies and Flow Cytometry

Blood cells procedures were initiated after obtaining informed consent from volunteer donors (n = 3). Mononuclear cells were isolated from EDTA-stabilized peripheral blood by centrifugation on Histopaque-1077 gradient (Sigma-Aldrich, USA). After 3-fold washing with phosphate buffer (Biolot, Saint Petersburg, Russia), the viability of mononuclear cells (Lym) was more than 98%. Lym was then transferred into 24-well plates (Corning, USA) at 10,000 cells per well with 3 mL of RPMI-1640 culture medium (Sigma-Aldrich, USA) containing 10% fetal serum (Stemcell, Vancouver, BC, Canada), L-glutamine (Stemcell, Canada) and gentamicin (Sintez, Kurgan, Russia). The next day, Lym in each well was resuspended and transferred to new 24-well plates with pre-positioned microfiber scaffolds (PLA scaffold group), forty days hiPSC-CM (CM group), or hiPSC-CM-on-scaffolds (TEC group). The negative control group included intact Lym in culture medium. For the positive control, Lym was incubated with 10 µg/mL concanavalin A (conA group) (Sigma-Aldrich, USA). Two days later, Lym was collected from the plates and fixed using 2% paraformaldehyde.

Cells were labeled with anti-human CD3-APC-A750, CD4-APC, CD8-APC-A700, CD28-PC5 and CD152-PE (CTLA-4) antibodies (Beckman Coulter, Indianapolis, IN, USA) at recommended concentrations. The stained Lym after washes was analyzed on a Navios flow cytometer set (Beckman Coulter, USA) using Kaluza software. Samples from each of the five experimental groups were examined twice.

### 2.4. Electronic and Fluorescence Microscopic Examinations

PLA samples for SEM were coated with a 10 nm gold layer in a Q150R automatic magnetron sputtering machine (Quorum Technologies, Lewes, UK). The morphology of the obtained PLA microfiber substrates was studied using a JSM-6510LA scanning electron microscope (JEOL, Tokyo, Japan).

Fluorescent visualization of live CMs at the stages of TEC fabrication was performed using a TMRM assay kit, antibodies to sarcomeric alpha-actin and Nkx2.5 (all Abcam, Cambridge, UK), according to the manufacturer`s recommendations. Generalized cardiomyocyte contraction on the scaffold was observed using Fluo-8-AM staining (Abcam, UK) and a Ti100 fluorescence microscope (Nikon, Tokyo, Japan) with Imstar software.

### 2.5. Ethical Statement

Experimental protocol was approved by Local Ethics Committee of «E. Meshalkin National Medical Research Center» of the Ministry of Health of the Russian Federation. (approval date, 26 December 2014, protocol 45).

### 2.6. Statistical Analysis

Statistical analysis was performed using Statistica 13 software (TIBCO Software, Palo Alto, CA, USA). The data were checked for normal distribution by the Kolmogorov–Smirnov test. Descriptive statistics are presented as mean ± standard deviation. Ordinary one-way ANOVA with Dunnet’s post hoc test was implemented to identify significant differences between groups. Values of *p* < 0.05 considered statistically significant.

## 3. Results

A protocol based on the WNT pathway activation by inhibiting the GSK3 enzyme with CHIR99021, followed by WNT repression (with IWP2), was applied to induce iPSC differentiation into cardiomyocytes. The first spontaneous contractile cells were detected on day 8 of the directed differentiation. During cell cultivation, the number of contractile sites and the intensity of contractions increased. The hiPSCCM metabolic selection method used provided a pure monolayer cell culture. Fluo-8-AM, a green fluorescent calcium-binding dye, was used to visualize ionic currents produced by the contraction of the СМ culture (Supplementary Materials Video S1).

**Figure 1:**
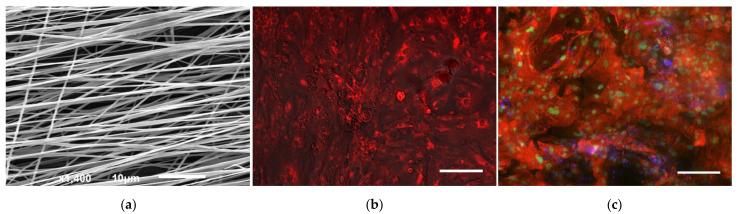
Bare and seeded PLA scaffolds: (**a**) SEM after electrospinning, bar 10 µm; (**b**) TMRM staining of TECs, bar 50 µm; (**c**) immunofluorescent staining of TECs for Nkx2.5 (green), sacromeric a-actin (red), nuclei (DAPI, blue), bar 50 µm.

Upon completion of metabolic selection, CMs were seeded onto PLA scaffolds. The electrospinning protocol used made it possible to obtain nonwoven PLA materials with aligned fibers. SEM analysis showed a fiber diameter of 0.5–1 µm (Figure 1a). The extracellular matrix Matrigel™ provided cell attachment and de novo formation of intercellular connections. A TMRM assay was used to assess viability after cell transfer to scaffolds by monitoring CM mitochondrial function. Under normal conditions, TMRM accumulates in negatively polarized mitochondria and has an emission maxima at 573 nm. In apoptotic or metabolically stressed cells, mitochondrial membrane potential drops, and the TMRM separates and spreads throughout the cytosol, resulting in a significant decrease in fluorescence intensity (Figure 1b). Immunofluorescence staining of the developed TECs confirmed the presence of characteristic markers of differentiated CM. Simultaneous staining for cardiomyocyte nuclear differentiation factor Nkx2.5 and sarcomeric alpha-actin was performed (Figure 1c).

On the second day of culturing the reseeded CM, a functioning and pulsating cardiac construct was obtained (Supplementary Materials Video S2).

Recall that hiPSC-CM was 25 days old by the end of metabolic selection. After that, a part of the cells was used for seeding PLA scaffolds, and the other part was cultured in culture medium. In both cases, it took 14 days. Thus, by the beginning of the immunological studies, the age of the hiPSC-CM in the CM and TEC groups was the same.

**Figure 2:**
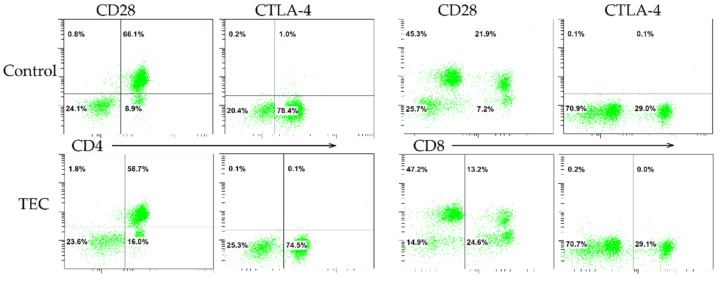
Expression of CD28 and CTLA-4 on CD4^+^ and CD8^+^ T lymphocytes induced by concanavalin A (control) or hiPSC-derived cardiomyocytes on PLA scaffolds (TEC).

Flow cytometric evaluation of the immune response activation regulators CD28 and CTLA-4 on T lymphocytes was carried out 48 h after co-cultivation of MNCs and experimental samples. Primary selection of lymphocytes from impurities (detached hiPSC-CM, other blood cells) was performed according to CD45 and CD3 markers. An analysis of T lymphocyte populations revealed no differences in the number CD3^+^/CD4^+^ and CD3^+^/CD8^+^ cells between the experimental groups. Approximately half of the CD4^+^ T lymphocytes in all groups (55–65%) expressed the activation marker CD28. CD8^+^ T lymphocytes expressed this marker three times less. CTLA-4 expression was less than 0.5% in experimental groups. Only upon stimulation with conA did the number of CD4^+^/CTLA-4^+^ T lymphocytes reach 1.2 ± 0.5% (Figure 2).

**Figure 3:**
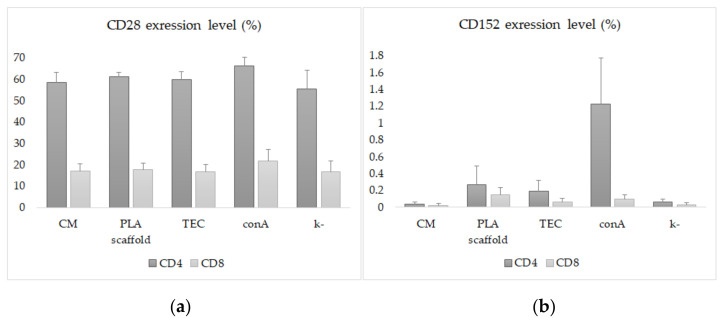
Flow cytometric analysis of CD28 (**a**) and CTLA-4 (**b**) expression on CD4^+^ and CD8^+^ T lymphocytes: CM—culture of hiPSC-derived cardiomyocytes; PLA scaffold—microfiber scaffold without CM; TEC—hiPSC-derived cardiomyocytes on PLA scaffold; conA—cell stimulation with concanavalin A; k-—intact cells in culture medium.

There was a statistically significant decrease in the expression levels of CD28 and CTLA-4 on T-lymphocytes in the CM, TEC and negative control groups compared to the positive control (*p* < 0,05). Concanavalin A stimulation slightly increased the expressions of CD28 and CTLA-4 on CD4^+^ and CD8^+^ T lymphocytes (Figure 3, Supplementary Materials Table S1).

Thus, our data show that the developed cardiac constructs and culture of human iPSCs-derived cardiomyocytes did not cause an increase in CD28 and CTLA-4 expression by human T-lymphocytes. On this basis, one can expect the absence of a recipient’s immune response to future transplantation of this tissue-engineered construct.

## 4. Discussion

In this work, we continued our earlier studies on the properties of hiPSC-CM cultured on biocompatible and biodegradable substrates [13,19]. The proposed methodological approach can be used to obtain spontaneously contracting cardiac cell colonies for further assembly of human myocardium fragments, biological cardiac pacemakers, cell organoids and other electrophysiologically active cell systems for pharmaceutics and regenerative medicine [20].

We used a previously published and standardized protocol for directed differentiation of CM from iPSCs [6]. Purification and enrichment of the CM population was performed using metabolic selection based on the ability of cardiomyocytes to metabolize lactate in the absence of glucose [21]. The selected method allows for the production of iPSCs-derived CM that have spontaneous contractile activity and express the main cardiac differentiation markers (Nkx2.5, sacromeric alpha-actin) [13,21].

TEСs were based on PLA scaffolds with unidirectionally oriented fibers arranged rather sparsely. Such a filament arrangement was better suited for the cultivation of contracting cells and assembly of cardiac patches [19]. Other researchers also reported the relationship between fiber alignment and the nature of cardiomyocyte function [22,23,24,25]. A study by Parrag I. et al. (2012) showed that aligned electrospun polyurethane scaffolds led to anisotropic organization of cardiomyocytes and improved their sarcomere formation [24]. The use of polymer microfiber scaffolds as substrates for the cultivation of iPSC-СМ allows for the controlling of the transformation of a group of cardiomyocytes into a consolidated structure [26,27]. This approach can provide the mechanical strength and functional electrophysiological unity of the cells within the TECs and leads to increase survival of tissue-engineered cardiac constructs cells after transplantation [13].

Transplantation techniques for tissue-engineered constructs carrying live cells are a separate field of research [28,29]. However, preparation for any transplantation requires predicting the probability of development and investigation of the mechanisms of post-transplant reactions both to the entire tissue-engineered construct and to its elements. For this purpose, it is necessary to perform immunological studies.

One of the earliest mechanisms of transplant immunity manifestation is the activation of the CD28/B7/CTLA-4–receptor complex on lymphocytes and antigen-presenting cells (APCs) [30,31,32]. Increased expression of CD28 and CTLA-4 on T-lymphocytes reflects the activation of the immune response. It is known that the interaction of the co-stimulatory molecule CD28 with B7 ligands (termed CD80/86) on the APCs leads to T cell activation. However, CTLA-4 binding to B7 induces T cell anergy [32]. The prevalence of one or another interaction in the process of antigen presentation determines the outcome: the formation of an active clone of T cells and the activation of transplant rejection mechanism or the formation of recipient tolerance [33]. An imbalance in the expression or hyperstimulation of CD28 has been realized either in the form of transplantation failures or in the development of autoimmune diseases [34,35]. Expression of CTLA-4 by immune cells reflects the formation of a feedback mechanism that limits the excessive activation of effector T lymphocytes. It is a marker of the negative regulation of the immune response. [36,37].

We found that iPSCs-derived cardiomyocytes cultured on PLA scaffolds did not increase the expression of immune response markers CD28 and CTLA-4 on T lymphocytes. Perhaps the achieved period of directed differentiation is not yet enough to form a full-fledged antigenic phenotype of cardiomyocytes and the triggering of the “friend-or-foe” recognition mechanism by recipient cells. That said, Säljö K. et al. (2017) showed that 26 days were enough for expression of different HLA antigens on the human pluripotent stem cell surface [38]. It is not yet known whether the fact that we used a different cell line influenced the result or whether there were minor differences in the directed differentiation protocols in our studies. However, another interesting fact should be noted.

Some researchers speculate about the absence of MHC-I class not only on iPSCs and iPSC-derived cardiomyocytes, but also on adult cardiomyocytes [38,39,40]. This controversial statement is based on works from the mid-1980s. We see it as a contradiction to the foundational principles of immune response formation and transplant immunity, evidence of which has been obtained experimentally and clinically proven in children and adults [14,41,42,43,44,45,46]. As it turned out, this aspect of pluripotent stem cell biology is poorly understood. Therefore, we plan to further investigate the expression of MHC antigens during the differentiation stages of iPSC-derived CM.

## 5. Conclusions

There remains high interest in cardiac tissue engineering, which creates scaffolds to control cell behavior, thereby restoring the natural architecture and function of various parts of the heart. The concept of in situ tissue engineering represents an innovation for creating a living and immunocompatible scaffold, which significantly reduces the time of implant preparation [47]. In this line of research, the great advances in laboratory studies of iPSCs explain the steady progress in cardiac tissue engineering in recent decades, and are probably the next focus in cardiac regeneration.

With this work, we demonstrate some immunological properties of a novel biodegradable microfiber scaffold that allows human iPSCs-derived cardiomyocytes to orient and contract. Neither PLA scaffolds carrying human iPSC-CM nor forty-day differentiated cardiomyocytes alone induce increased expression of the early T cell activation markers CD28 and CTLA-4. A continuation of the studies will be the transplantation of cardiac TECs into rats and pigs with an assessment of the electrophysiological and histological outcomes of these interventions.

## Supplementary Materials

The following are available online at https://www.mdpi.com/article/10.3390/jfb13010006/s1, Video S1: Oscillation of calcium ions during generalized contraction by hiPSC-CM culture; Video S2: Oscillation of calcium ions by hiPSCs-CM on a PLA scaffold; Table S1: Profile of CD28 and CTLA-4 expression on T-lymphocytes.
