# Supplementary material for: Do Human iPSC-Derived Cardiomyocytes Cultured on PLA Scaffolds Induce Expression of CD28/CTLA-4 by T Lymphocytes?
Source: J Funct Biomater. 2022 Jan 11;13(1):6. doi: 10.3390/jfb13010006 (PMC8788528; doi:10.3390/jfb13010006)
Supplement: Supplementary file 1 [file jfb-13-00006-s001.zip › Table S1.pdf]

Do Human iPSC-Derived Cardiomyocytes Cultured on PLA Scaffolds Induce Expression of CD28/CTLA-4 by T Lymphocytes?

David Sergeevichev, Victor Balashov, Victoria Kozyreva, Sophia Pavlova, Maria Vasiliyeva, Alexander Romanov and Elena Chepeleva

Table S1. Profile of CD28 and CTLA-4 expression on T-lymphocytes

Group	$CD4^+/CD28^+$	$CD4^+/CD152^+$	$CD8^+/CD28^+$	$CD8^+/CD152^+$
CM	58,72 ± 4,54	0,04 ± 0,03	17,21 ± 3,92	0,02 ± 0,02
PLA scaffold	61,35 ± 1,80	0,27 ± 0,23	17,87 ± 2,83	0,15 ± 0,09
TEC	59,87 ± 3,80	0,19 ± 0,13	16,64 ± 3,60	0,06 ± 0,05
conA	66,31 ± 1,56	1,23 ± 0,55	21,70 ± 5,33	0,10 ± 0,05
neg control	55,48 ± 8,85	0,06 ± 0,03	16,85 ± 5,04	0,03 ± 0,01

CM – culture of hiPSC-derived cardiomyocytes; PLA scaffold - microfiber scaffold without CM; TEC - hiPSC-derived cardiomyocytes on PLA scaffold; conA – cell stimulation with concanavalin A; k- – intact cells in culture medium.
